# Ovarian Tumor Microenvironment Signaling: Convergence on the Rac1 GTPase

**DOI:** 10.3390/cancers10100358

**Published:** 2018-09-27

**Authors:** Laurie G. Hudson, Jennifer M. Gillette, Huining Kang, Melanie R. Rivera, Angela Wandinger-Ness

**Affiliations:** 1 Department of Pharmaceutical Sciences, University of New Mexico Health Sciences Center, Albuquerque, NM 87131, USA; 2 Comprehensive Cancer Center, University of New Mexico Health Sciences Center, Albuquerque, NM 87131, USA; jgillette@salud.unm.edu (J.M.G.); HuKang@salud.unm.edu (H.K.); MelRivera@salud.unm.edu (M.R.R.); awandinger-ness@salud.unm.edu (A.W.-N.); 3 Department of Pathology, University of New Mexico Health Sciences Center, Albuquerque, NM 87131, USA; 4 Department of Medicine, University of New Mexico Health Sciences Center, Albuquerque, NM 87131, USA

**Keywords:** Rho-GTPase, Rac1, guanine nucleotide exchange factors (GEFs), GTPase activating proteins (GAPs), oncogene, oncoprotein, ovarian cancer, tumor microenvironment, bone niche, therapeutic targeting

## Abstract

The tumor microenvironment for epithelial ovarian cancer is complex and rich in bioactive molecules that modulate cell-cell interactions and stimulate numerous signal transduction cascades. These signals ultimately modulate all aspects of tumor behavior including progression, metastasis and therapeutic response. Many of the signaling pathways converge on the small GTPase Ras-related C3 botulinum toxin substrate (Rac)1. In addition to regulating actin cytoskeleton remodeling necessary for tumor cell adhesion, migration and invasion, Rac1 through its downstream effectors, regulates cancer cell survival, tumor angiogenesis, phenotypic plasticity, quiescence, and resistance to therapeutics. In this review we discuss evidence for Rac1 activation within the ovarian tumor microenvironment, mechanisms of Rac1 dysregulation as they apply to ovarian cancer, and the potential benefits of targeting aberrant Rac1 activity in this disease. The potential for Rac1 contribution to extraperitoneal dissemination of ovarian cancer is addressed.

## 1. Introduction

Despite advances in treatment, long-term outcomes for epithelial ovarian cancer (EOC) patients remain discouraging. Challenges to effective treatment include factors such as diagnosis after tumor dissemination, presence of residual disease after treatment, a limited number of identified targets for maintenance therapy, and acquired chemoresistance leading to relapse after initial clinical remission [1,2]. EOC displays a high degree of genomic heterogeneity [3,4] and it has been proposed that tumor microenvironmental factors may also contribute to tumor heterogeneity [5].

EOC dissemination occurs predominantly through tumor cell exfoliation into the peritoneal cavity thereby providing a unique environment for tumor growth and metastasis when compared to the majority of solid tumors [6,7,8,9,10]. There is heterogeneity of sites within the peritoneal cavity leading to diverse localized environments. For example, the omentum is rich in adipocytes and provides a distinct niche when compared to the mesothelium of the peritoneal wall [10,11,12,13,14,15,16,17,18]. Furthermore, the tissue underlying the mesothelial lining at various locations differs in architecture and local production of chemotactic factors thus promoting different adhesive and invasive behaviors [11]. It may be more accurate to consider the peritoneal cavity as home to multiple tumor microenvironments (TMEs) presenting additional challenges to effective treatment.

**Figure 1:**
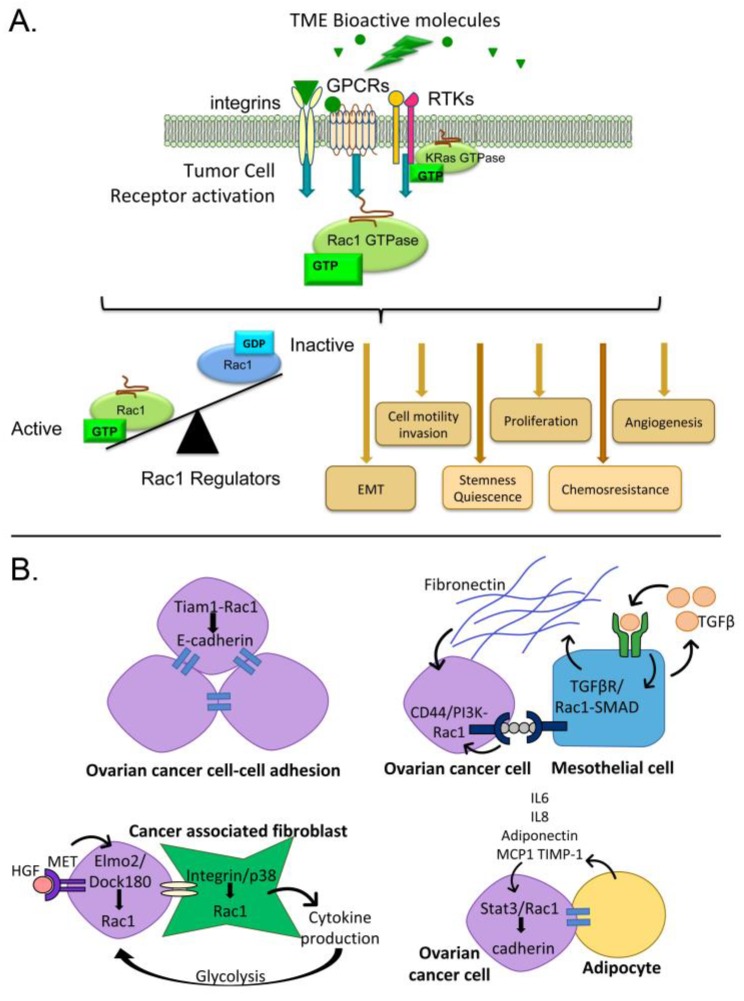
Bioactive molecules in the tumor microenvironment (TME) activate multiple receptors that converge on Rac1. (**A**) Examples of receptors that activate Rac1 in response to bioactive molecules in the TME are shown (GPCR = G protein-coupled receptor; RTK = receptor tyrosine kinase). Rac1 activity is balanced through multiple regulatory mechanisms discussed in this review that serve to control diverse physiological outcomes. (**B**) Cell-cell interactions between tumor cells themselves or with cells in the TME (adipocytes, cancer-associated fibroblasts, mesothelia) can also cause Rac1 activation and further modulate the TME. See Section 3.3 for further detail.

Tumor cells within the ovarian cancer TME are exposed to a variety of regulatory signals. Tumor cells interact with mesothelium, fibroblasts, endothelium, immune cells and other cells in the TME [6,7,8,9,10,19,20]. Invasive cells come into contact with the extracellular matrix (ECM) underlying the mesothelium. This leads to intracellular signaling due to integrin engagement and exposure to ECM-associated growth factors. Each cell type in the TME, as well as the tumor cells themselves, secrete bioactive molecules that accumulate in the peritoneal fluids and drive adverse tumor cell behaviors such as proliferation, invasion, and phenotypes promoting chemoresistance. These cell-cell interactions between tumor cells or other cells in the TME, cell-matrix interactions, and exposure to growth factors and cytokines present in peritoneal fluids all stimulate signaling cascades that dictate aspects of tumor cell function. Many of these diverse signals converge upon, and are integrated through, the small GTPase Ras-related C3 botulinum toxin substrate (Rac) 1 (Figure 1) [21,22,23,24,25,26].

The Rac subfamily of Rho family small GTPases has three members. Rac1 is the best-characterized member of this subfamily with strong evidence for Rac1 dysregulation in cancer [21,23,24,25,27,28]. Rac2 expression is confined to hematopoietic cells [29] and Rac3 has not been studied in the context of ovarian cancer so these two proteins will not be discussed further in this review. Rac1 acts as a molecular switch by cycling between active and inactive states that depend upon nucleotide binding (Figure 1A) and other regulatory mechanisms discussed below. As a focal point for multiple signaling pathways, Rac1 is capable of shunting cells between proliferation, apoptosis or quiescence, altering cell differentiation and transcription, and modulating cell-environment interactions. Based on the known activities of Rac1, this protein can play important roles in multiple steps of tumor development, dissemination and disease recurrence.

## 2. Consequences of Rac1 Activation in Cancer

Rac1 cycles between an active GTP-bound state and an inactive GDP-bound conformation (Figure 1A) regulated by guanine nucleotide exchange factors (GEFs), GTPase-activating proteins (GAPs) and guanine nucleotide dissociation inhibitors (GDIs) [24]. Aberrant activation of Rac1 is implicated in numerous aspects of tumor development and progression and is the subject of several recent reviews [25,26,30,31]. Rac1 is best recognized for translating extracellular signaling into downstream changes in actin remodeling, cell adhesion, motility and invasion [23,26,32]. There is strong emerging evidence that Rac1 also contributes to the tumor stem cell phenotype, epithelial to mesenchymal transition (EMT), angiogenesis, and chemoresistance [33,34,35,36,37,38]. Elevated Rac1 activity is associated with enhanced stem cell characteristics in multiple cancers and its inhibition attenuates the stem cell phenotype [34,36,39]. Although the relationship between Rac1 expression and/or elevated activity and cancer stem cells has been reported for several cancer types, there is little information for ovarian cancer. However, a splice variant of Vav3, a GEF and enhancer of Rac1 activity, is overexpressed in multi-drug resistant stem cell-like fractions of ovarian cancer cells [40]. This finding suggests that elevated Rac1 activity may promote stem cell characteristics in ovarian cancer similar to the reports for other tumor types.

Ovarian cancer cells display phenotypic plasticity with gains and losses of epithelial characteristics during tumor development and peritoneal metastasis [41,42,43]. EMT is viewed as a critical aspect of tumor invasion and metastasis [44,45] and Rac1 is implicated in promoting EMT in a number of cancers [25]. Experimental evidence demonstrates that elevated Rac1 activity is sufficient to drive aspects of EMT in ovarian tumor cells. When a mutationally activated form of Rac1 (Rac1G12V) was introduced into ovarian tumor cells with an epithelial phenotype, cells displayed morphologic characteristics of EMT including down-regulation of the epithelial marker E-cadherin, up-regulation of the mesenchymal marker vimentin, and increased invasive capacity [46]. Inhibition of Rac1 activity or knockdown of Rac1 expression restored epithelial characteristics to ovarian tumor cells [13,46,47] and inhibited migration and invasion [47,48,49]. The significance of EMT in ovarian cancer is demonstrated by the presence of ovarian cancer cells in extraperitoneal sites [50,51,52] and the circulation [50,53,54,55] where the circulating tumor cells display mesenchymal characteristics [54,56]

Tumor angiogenesis supplies necessary nutrients and fosters tumor growth [22]. Angiogenesis is a critical aspect of ovarian cancer and this process is targeted by therapeutics in current care [57,58,59]. Rac1 is involved in angiogenesis and required for vascular integrity and blood vessel sprouting as demonstrated in a conditional Rac1 knockout mouse model [60]. In humans, Rac1 expression correlated with blood vessel invasion in a meta-analysis of multiple cancer studies [38]. Rac1 is activated by the angiogenic factors vascular endothelial growth factor (VEGF)-A, angiopoietin 1, basic fibroblast growth factor (FGF) and others [61]. Activation of Rac1 in endothelial cells regulates adhesion, filopodia, morphogenesis, cell proliferation and migration [33,62,63,64,65]. Two different Rac1 inhibitors displayed anti-angiogenic activity in breast cancer models in vivo [66,67] supporting a potential benefit of Rac1 inhibition as an alternate anti-angiogenic strategy in cancer, including ovarian cancer.

Lymphangiogenesis is driven by the VEGF-C ligand and its high affinity receptor VEGFR3 [68,69]. The well-established omental niche site has vessels that express high levels of the neoangiogenic VEGFR3, which serve to recruit ovarian tumor cells and offer a supportive environment for neovascularization [70]. High VEGF-C expression is associated with worse overall survival in ovarian cancer patients and tumor cell expression of VEGF-C is critical for lymphatic invasion and lymphangiogenesis [71,72]. Mechanistic studies show VEGF-C signaling to Rac1 requires VEGFR3 endocytosis mediated by EphrinB2 [73]. In colorectal and lung cancers, lymph node metastasis mediated by VEGF-C is linked to high expression of the Rac1 activating GEF Tiam1 [74,75]. Conversely, a chemical library screen identified statins as potent inhibitors of lymphangiogenesis by blocking Rac1 prenylation and plasma membrane recruitment [69]. In this regard it is worth noting that inhibition of VEGFR3 signaling in OVCAR8 cells, via Maz51, induced chemosensitization through downregulation of BRCA gene expression. This finding suggests that combined targeting of VEGFR3 and Rac1 may have benefit for dually blocking metastasis and enhancing tumor cell killing [76].

Rac1 is gaining substantial attention as a mediator of chemoresistance [37,77,78]. Rac1 is implicated in treatment resistance in multiple cancers [37] and Rac1 inhibition increases sensitivity to doxorubicin for squamous cell carcinoma cells, 5-fluorouracil and cisplatin in gastric adenocarcinoma spheroids, and fludarabine for chronic lymphocytic leukemia (reviewed in [37]). In addition to these conventional chemotherapies, Rac1 is suspected in resistance to a number of targeted therapies through regulation of compensatory mechanisms. These include therapies directed against the epidermal growth factor (EGF) receptor and human epidermal growth factor receptor (HER)-2 for lung and breast cancers, B-RAF protein inhibitors in melanoma, estrogen targeted therapies in breast cancer and VEGF/VEGFR targeted therapies in prostate cancer (reviewed in [30]). In many cases sensitivity to the targeted therapeutic is restored upon Rac1 inhibition. The contributions of Rac1 activation to chemoresistance is likely multifaceted based on specific mechanisms along distinct drug action pathways, as well as non-specific mechanisms related to Rac1 promotion of EMT and stem cell characteristics [42,79,80,81].

## 3. Pathways for Rac1 Activation by the Ovarian Tumor Microenvironment

Extracellular signals mediated by various cell surface receptors such as integrins, cadherins, cytokine receptors, G-protein-coupled receptors (GPCRs) and receptor tyrosine kinases (RTKs) activate GEFs and recruit Rac1 (sequestered with GDIs) from the cytosol to the plasma membrane or other cellular locations (Figure 3A [21,30,82,83]). Rac1 then activates effector molecules including proteins involved in actin remodeling, kinases, and adapter proteins that are responsible for propagating Rac1-dependent signals and subsequent biological responses. The specific stimulus can dictate distinct responses to Rac1 activation based on post-translational modifications of Rac1, GEFs or other Rac1 modulatory molecules or effectors [21]. Because Rac1 is responsive to an array of signals, Rac1 is capable of driving multiple steps of tumor development, dissemination and recurrence. A few examples of Rac1 activation by common components of ascitic fluids are described in more detail below.

### 3.1. Activators of G-Protein Coupled Receptors and Rac1 Activity

The bioactive lipids lysophosphatidic acid (LPA) and sphingosine-1-phosphate (S1P) are present in ascitic fluid of ovarian cancer patients and activate GPCRs upstream of Rac1. Elevated levels of LPA and S1P are both associated with ovarian tumor cell migration, invasion and metastasis [6,84] and these processes require Rac1-dependent actin remodeling. Pharmacologic inhibition of Rac1 decreased S1P-dependent ovarian tumor cell invasion [85]. When multiple ovarian tumor cell lines were studied, the ability of LPA to stimulate migration was highly correlated with LPA-dependent Rac1 activation [86]. Expression of a dominant negative form of Rac1 ablated LPA-stimulated cell migration and in vivo metastatic colonization in responsive cell lines. Conversely, expression of a constitutively active form of Rac1 conferred migration and in vivo implantation to cell lines non-responsive to LPA [86]. Knock-down strategies determined that a Rac1-activating SOS1/EPS8/ABI1 complex unique to metastatic cells was responsible for the LPA stimulated migration and invasive implantation in mice [86]. LPA activation of Rac1 has also been reported to be dependent on a Src/p130Cas pathway for ovarian cell migration [87] and the Rac1 GEF βPIX was necessary for LPA-induced invadopodia formation [88] although βPIX knock-down did not disrupt LPA-stimulated migration in certain ovarian tumor cell lines [86]. The reported observations indicate that distinct Rac1 regulatory mechanisms are responsible for different functional outputs and there may be cell-specific differences based on the expression or activity of Rac1 regulators.

### 3.2. Activators of Tyrosine Kinases and Rac1 Activity

Ligands for RTKs such as the EGF receptor and VEGF receptor are prevalent in ovarian cancer ascites and regulate Rac1 activation through multiple mechanisms. Signaling through RTKs activate phosphatidylinositol-3 kinase and phospholipase C-γ to modulate targeting of Rac1 regulatory proteins such as GEFs and GAPs and recruit GEFs to signaling complexes through post-translational modifications (reviewed in [21,89]). In certain cases, signaling receptors can modify Rac1 activity directly. For example, EGF receptor-stimulated ERK phosphorylation of Rac1 on T108 targets Rac1 for nuclear translocation [21]. Rac1 has been shown to be an essential component of EGF receptor signaling in different tumor types [90,91] and implicated in EGF receptor driven tumorigenesis [91]. Ligands present in the ovarian TME are likely to activate Rac1 by impinging on ErbB3, ErbB4 and MET receptors, which are expressed in 76–98% of ovarian tumors [92]. For example, heregulin stimulation of ErbB3 and ErbB4 causes upregulation of C-X-C chemokine receptor type 4 (CXCR4) and increases Rac1 activation through a stromal cell-derived factor (SDF)-1-CXCR4 mediated PREX1 GEF mechanism in breast cancer cells [93]. Hepatocyte growth factor (HGF) induces a MET-AXL-ELMO2-DOCK180 complex that activates Rac1-dependent cancer cell migration and invasion [94]. Pharmacologic inhibition of Rac1 inhibited EGF-stimulated p21-activating kinase (PAK) phosphorylation, filopodia formation and invadopodia [48,95] in ovarian tumor cell lines indicating contributions of Rac1 in cancer-relevant functions. Although specific mechanisms of Rac1 activation by VEGF have not been explored in ovarian cancer models, there is abundant evidence that Rac1 is a component of VEGF signaling to angiogenesis. Ablation of Rac1 in endothelial cells in development is embryonic lethal due to lack of neovascularization [96]. Studies show that Rac1 activation is critical for normal in vivo angiogenesis in adult mice due to junctional stabilization required for mature vessels [97]. More recent work indicates that lumen formation and stable cell:cell contacts are mediated through the GEF DOCK4 activation of Rac1 [62]. The combined data indicate that further study of Rac1 activation in ovarian cancer by tyrosine kinase receptors and their interfaces with G-protein coupled receptors is warranted.

### 3.3. Cell Interactions Leading to Rac1 Activation

An article in the present series and other recent reviews provide an in depth analysis of cell-cell interactions in the ovarian tumor microenvironment that drive ovarian cancer progression [9,57,98]. Here, we briefly highlight how some of these interactions may promote ovarian cancer metastasis through Rac1-dependent mechanisms (Figure 1B).

#### 3.3.1. Tumor Cell-Cell Adhesion

Rac1 signaling is important for cell-cell adhesion. Ovarian cancer cells in the ascites fluid form multicellular aggregates (spheroids) that facilitate angiogenesis and invasion of various peritoneal organs [11]. Tumor cell-cell adhesions are mediated by E-cadherin maintenance of cell-cell junctions that depend on a Rac1-Tiam1 GEF-IQGAP1 effector complex and promote an anti-migratory phenotype [99]. Ovarian cancer spheroids with high E-cadherin expression are less sensitive to cisplatin treatment suggesting an important role for cell-cell adhesions in spheroid chemoresistance [100].

#### 3.3.2. Mesothelial Cells

Ovarian cancer frequently metastasizes to the peritoneal wall, which is lined with mesothelial cells. Ovarian cancer cell interactions with mesothelial cells can stimulate mesothelial cell production of fibronectin through the autocrine secretion of transforming growth factor (TGF)-β1. This activates a TGF-βR1/Rac1/SMAD-dependent signaling pathway in mesothelial cells. The activated mesothelial cells and production of fibronectin contributes to metastasis by supporting tumor cell adhesion, invasion, and proliferation [13,57]. Co-culture of ovarian cancer cell lines with mesothelial cells led to upregulated expression of the hyaluronan receptor and stem cell marker CD44 and promoted tumorigenesis in a xenograft model [101]. CD44 promotes ovarian tumor cell-peritoneal cell adhesion through binding of its ligand hyaluronan in complex with versican [102] and is generally known to signal through multiple pathways downstream of Rac1 to promote tumor cell invasion [103].

#### 3.3.3. Fibroblasts

Ovarian tumor cell-fibroblast interactions cause conversion of normal fibroblasts to cancer-associated fibroblasts (CAFs, distinguished by smooth muscle actin expression) and lead to increased tumor cell adhesion and overexpression of HGF and matrix metalloproteinase (MMP) [104]. MET receptor activation by HGF induced recruitment of the bipartite Rac1 GEF Elmo2/Dock180 and promoted Rac1-dependent migration and invasion of multiple cancer cell lines in vitro, though ovarian cell lines were not specifically tested [94]. Interactions between human omental CAF and ovarian tumor cells also result in an integrin/p38/Rac1-dependent activation of cytokine secretion by CAFs, which in turn promotes tumor cell proliferation and metastasis through activated glycogen breakdown and glycolysis [105].

#### 3.3.4. Adipocytes

The omentum is a favored ovarian tumor cell niche based on initial chemoattraction by adipocyte secreted factors that can stimulate Stat3-mediated Rac1 activation [106]. In turn, the activation of these pathways can strengthen cadherin-dependent binding of tumor cells, provide tumor cells with an energy source through mutual changes in lipid metabolism, and promote invasion [14,106].

The selected illustrations do not capture the entire scope of potential ovarian cancer TME regulation of Rac1 activity. Inflammatory cytokines such as interleukins 6 and 8, tumor necrosis factor (TNF) α, and TGFβ are among the additional soluble factors in ascites fluids that are associated with worse prognosis and variously associated with proliferation, metastatic spread, angiogenesis, EMT and treatment resistance [6,107]. Each of these bioactive molecules is capable of stimulating signaling cascades leading to Rac1 activation through direct or indirect mechanisms [24]. In addition, integrin engagement and focal adhesion kinase activation recruits Rac1 to regulate spreading and adhesion on the extracellular matrix [26,89,108]. Immune cells are an integral part of the ovarian cancer TME and perform immune suppressive and activating functions that are pivotal in disease pathology [109,110] and these cells serve as important therapeutic targets [111,112]. The best-studied example of immune cell coupling to Rac1 activation in ovarian cancer is through cytokine activation of CXCR4 as detailed in Section 4.2 and Section 6. A more complete understanding of the complexities of Rac1 regulation by the ovarian cancer TME will require further study.

## 4. Mechanisms of Rac1 Dysregulation and Evidence in Ovarian Cancer

We reported that Rac1 protein is overexpressed and hyperactivated in ovarian cancer patient samples [113]. Addressing the function of Rac1 hyperactivation in ovarian cancer is an important research area because of the known roles of Rac1 in cancer metastasis and recurrence. In cancer, Rac1 is frequently released from normal control mechanisms through mutation [114,115,116,117,118], aberrant regulation of nucleotide binding and hydrolysis [26,30,119], and altered splicing [120,121,122,123,124,125,126,127,128,129,130]. Insight into possible mechanisms leading to Rac1 overexpression and hyperactivation in ovarian cancer is garnered from analyses of the Catalogue of Somatic Mutations in Cancer (COSMIC) and The Cancer Genome Atlas (TCGA) databases as detailed below.

### 4.1. Rac1 Overexpression and Somatic Mutation

**Figure 2:**
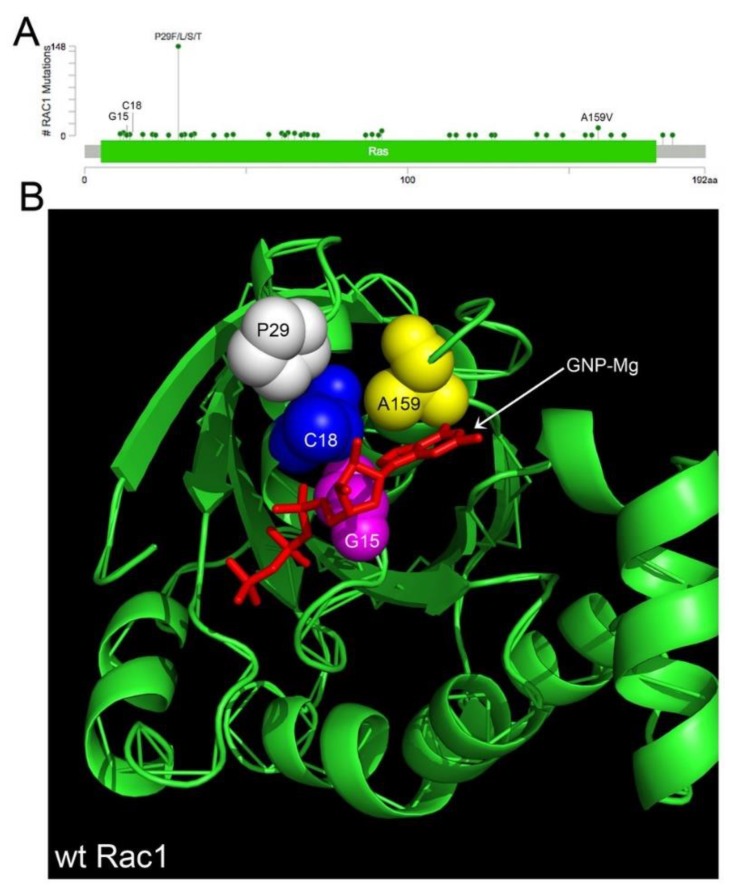
Pathogenic Cancer Mutations in Rac1. Rac1 gain of function mutations occur with low frequency (0.2–1%) in multiple cancer types, though as yet none have been found in serous ovarian cancer. (**A**) There are 239 pathogenic mutations in Rac1, resulting in missense substitutions at 46 amino acid residues. Melanoma has the highest frequency of Rac1 mutations, leading to substitutions at proline 29 and constitutive activation through GEF-independent fast nucleotide exchange. (**B**) Thirteen of the missense mutants are likely oncogenic (G12R/V/E, G15S, C18S/Y, P29F/L/S/T, Q61R/K, A159V) evidenced by recurrence at hotspots, paralogous with oncogenic mutations in Ras, or affecting residues that are clustered in the 3D structure close to the nucleotide binding site. Shown is the proximity of 4 point mutants in the crystal structure of wild-type Rac1 (PDB 3th5) rendered with MacPyMOL: PyMOL v1.5.0.5 (Schrödinger LLC).

There are 239 pathogenic missense mutations across diverse cancer types affecting 46 of the 192 amino acids in RAC1 (COSMIC v86 database updated in August 2018, https://cancer.sanger.ac.uk/cosmic/download). The mutants are clustered in conserved residues relevant to GTPase activity or affect residues close in 3D space that are important to Rac1 function (Figure 2A,B [117,118,131,132,133]). Select point mutants are the primary cause of constitutive Rac1 activation in some cancer types (melanoma, lung and germ cell cancers) (Figure 2A [114,115,116,117,118,132,134]). The highest prevalence (9%) of the constitutively active, fast cycling P29S mutant is found in melanoma [115]. To date, the functionally characterized Rac1 missense mutants (P29S, A159V, C18S and G15) all increase Rac1 activation and possibly expression [118,135]. Rac1 is not found mutant in the 315 serous ovarian cancer patient samples in the TCGA. However, given the low frequency of Rac1 missense mutants (0.01–0.02% for G15, C18 [118]) such rare mutations would be undetectable in the sample size and should not be taken as lack of evidence for the importance of Rac1 in ovarian cancer. For example, an shRNA essentiality screen of 29 ovarian cancer cell lines showed SKOV3, COV362, JHM + OM1 and SNU840 to have significantly decreased growth fitness with the loss of Rac1 (Harmonizome Achilles [136,137]. As another case in point, Rac1 is overexpressed due to gene amplification or mRNA upregulation in 21% (66/316) of the primary tumors in TCGA [138]. Despite the low frequency of RAC1 gene mutations, RAC1 is similar to well-known oncogenes and tumor suppressors in being categorized as a Tier 1 cancer-causing gene in the COSMIC cancer gene census. Therefore, further systematic study of the 239 Rac1 missense mutations is warranted. In contrast to tumor suppressor genes, where truncating mutations are prevalent and cause loss of function, the Rac1 mutations are like those in the oncoprotein Ras. The mutations appear in hotspots and tend to be activating mutations [139]. Thus, RAC1 is a Tier 1 cancer-causing gene and the mutational patterns in Rac1 are similar to many well-known oncogenes which are positive drivers of cancer.

### 4.2. Rac1 Regulators

**Figure 3:**
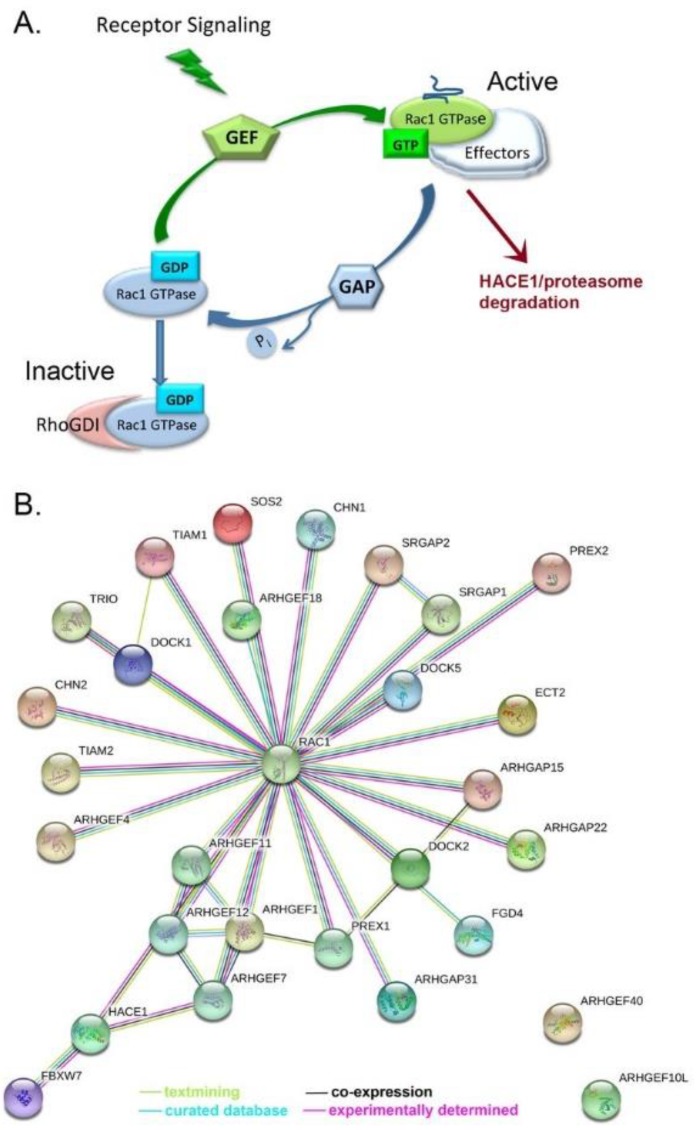
Rac1 Regulatory Network in Ovarian Cancer. Rac1 integrates signals downstream of tyrosine kinase receptors, adhesion molecules and G-protein coupled receptors and has nuclear functions. (**A**) There are over 40 GEF and GAP proteins involved in the regulation of Rac1 activity through the stimulation of nucleotide binding (GEFs) and hydrolysis (GAPs). Activated Rac1 binds to effectors that recognize the unique conformation of the GTP-bound GTPase and mediate downstream physiologic responses to receptor signaling. Activated Rac1 is ubiquitinated by HACE1 or FBXW7 and targeted for proteasomal degradation, further controlling protein levels and activity. (**B**) Twenty-six Rac1 GEF and GAP proteins, HACE1 and FBXW7 ubiquitin ligases have somatic mutations in serous ovarian cancer patient samples. String network analysis places Rac1 at the core with a large array of close functional associations with Rac1 regulatory proteins whose functions in ovarian cancer largely remain to be determined.

**Figure 4:**
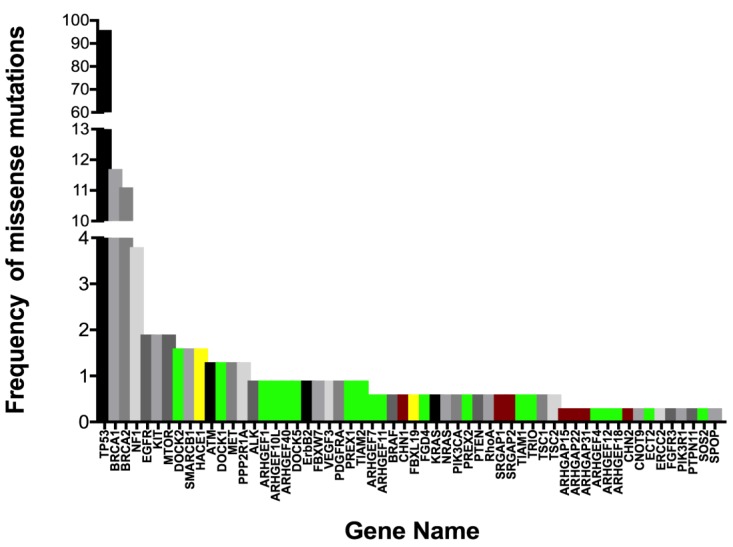
Rac1 regulators and effectors are part of the long tail of oncogenic drivers in ovarian cancer. A list of 54 genes with frequent missense mutations in cancers was derived from OncoKB: A precision oncology knowledge base and two recent publications on rare mutations. The cancer gene list was combined with a selected list of 28 Rac1 regulatory proteins (GEFs, GAPs, ubiquitin ligases). Plotted is the frequency of missense mutations in genes with mutation frequencies above 0 (58 of 82 analyzed) among 315 serous ovarian cancer patient samples in TCGA. The frequency for BRCA1 and BRCA2 gene mutations is the sum of somatic and germline missense mutations. For all other genes no germ line mutations are reported. Among the analyzed genes, 53 (9.2%) are Tier 1 of 574 reported in COSMIC v86; Tier 1 genes have “*documented activity relevant to cancer, plus evidence of mutations in cancer, which change the activity of the gene product in a way that promotes oncogenic transformation*”. Among the Rac1 regulators only ARHGEF12 and ARHGEF10L are validated as Tier 1 and Tier 2 (“*strong indication of a role in cancer*”), respectively. Cancer genes (black and gray), Rac1 GEFs (green), Rac1 GAPs (red), ubiquitin ligases (yellow). The data show that even though missense mutations in individual Rac1 regulators occur with low frequency, there are at least 26 possible targets (10 with co-occurring alterations, *p* < 0.05) that might lead to Rac1 activation or inactivation in ovarian cancer.

The activity of Rac1 is tightly controlled through a large network of GEF and GAP regulatory factors (Figure 3A,B [30,131,140,141,142,143]). This network is much greater than most other Ras-related GTPases. Rac1 GEF and GAP regulatory factors are mutant or exhibit altered expression in ovarian serous adenocarcinoma with a frequency of 0.3–1.6% based on our analyses of 28 relevant regulatory proteins in COSMIC v86 [144] and the cBioPortal platform for TCGA data viewing [19,131] (Figure 4 [20,83,118,138,144,145,146,147]).

Notably, the regulatory protein mutants show a high level of concurrent expression in tumor cells, suggesting that hitting multiple nodes releases key Rac1-regulated pathways from normal control. Even while the identified mutations often lie in known GEF and GAP regulatory domains, as well as in lipid or protein interaction domains, no systematic analyses have been completed to identify hotspot mutations or determine their pathogenicity in ovarian cancer. Nevertheless, some insights can be drawn from a handful of analyses of regulatory protein overexpression [109,148,149], truncation [150] or altered splice variants [40]; see also review [30].

Overexpression of the Rac1 GEF DOCK180 drives glioblastoma invasion through the activation of a Rac1-dependent kinase pathway [149]. A truncating mutant of PREX2 in melanoma has increased Rac1 GEF activity, and activates PI3K/AKT signaling, while abolishing binding to the PTEN tumor suppressor in melanoma [150]. An N-terminally truncated splice variant of the Vav3 GEF (Vav3.1) is a predictor of poor prognosis and platinum-response and highly expressed in ovarian cancer stem-like cell populations isolated from established cell lines [40]. These examples are supportive of a requirement for Rac1 activation in multiple cancers. Recent analyses of the metastatic TME using omental samples from patients with high grade serous ovarian cancer characterized secreted, matrix and cellular components [109]. Multivariate regression analyses of data were used to model the relationships between all TME components. Comprehensive RNA seq analysis of the TME identified 31 Rac1 GEFs, GAPS and ubiquitin ligases significantly associated with disease score by Pearson’s and Spearman’s tests; five GEFs and GAPs were significant based on Pearson’s only (supplementary Table 13 in [109]). Recent analyses of a large cohort of Canadian ovarian cancer patients identified variants in ARHGEF10L to be significantly associated with invasive disease [151] and three somatic missense mutations have been identified in ovarian cancer patient samples (COSMIC v86). The limited information on ARHGEF10L suggests in vitro GEF activity for RhoA, but not Rac1 or Cdc42 [152]. Since RhoA and Rac1 are often reciprocally active, connections between the two GTPases may need further analysis in ovarian cancer. Alterations in GAP expression in vivo have both activating and inhibitory effects on tumorigenesis and metastasis, likely due to dual roles as scaffolding proteins and GTP hydrolysis regulators [30]. When considering how to tackle prioritization of GEF and GAP proteins for study, categorizing potential tumor suppressive vs. promoting activity might be gained by using a ratiometric analysis of truncating/frameshift vs. missense mutations [139]. Additionally, functional analyses of select point mutants in key regulatory domains is an essential complementary effort that is necessary to understand effects on regulatory protein activity and pathway interconnections. The composite data are suggestive that Rac1 hyperactivation is an important driver in ovarian cancer and may result largely from the misregulation of GEF and GAP regulatory cascades rather than through activating mutations in Rac1 itself.

Emerging evidence suggests that Rac1 regulatory proteins function in spatially localized molecular assemblies. Such assemblies restrict Rac1 activity temporally and spatially to specific subcellular domains, which in turn restricts what downstream pathways are triggered by Rac1. In ovarian cancer, a recently described tripartite complex that includes the SOS1 GEF is essential for LPA-mediated Rac1 activation and metastasis [86]. Activation of Rac1 by the Tiam1 or PREX1 GEF proteins is spatially distinct in the cell and dictates anti- or pro-migratory responses in ovarian cancer cells [99]. The translocation of Rac1 in response to signaling and transient assembly of Rac1 GEFs at the plasma membrane can also occur through specific actin and protein based recruitment [82]. On the other hand, Rac1 forms a stable plasma membrane complex with CXCR4 independent of GTP-bound status, which is important for maintaining CXCR4 in a signaling competent conformation [153]. The PREX1 GEF is speculated to enable rapid response of Rac1 activation downstream of CXCR4 signaling. Therefore, functional studies of Rac1 and associated regulatory factors in the ovarian metastatic cascade will need to carefully consider spatiotemporal organization.

### 4.3. Rac1b Splice Variant

**Figure 5:**
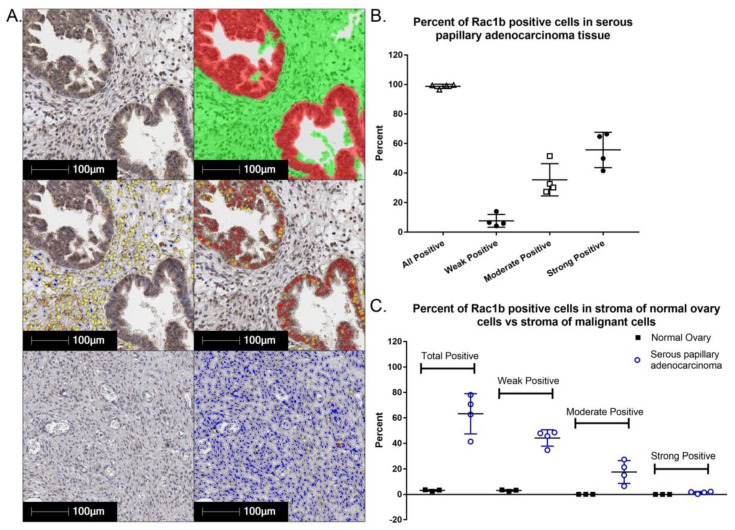
The constitutively active Rac1b splice variant is overexpressed in ovarian cancer. Ovarian cancer tissue microarrays were stained for Rac1b, a constitutively active Rac1 splice variant. Slides were imaged using an Aperio slide scanner and analysis was performed using HALO software. (**A**) Top panel: Malignant tissue stained with DAB for Rac1b. Analysis to identify tumor cells (red) and stromal cells (green). Middle panel: Quantification of the amount of Rac1b expression in tumor cells (right) vs. stromal cells (left) in malignant tissue. Blue-no staining, yellow-weak staining, orange-moderate staining, red-strong staining. Bottom panel: Quantification of Rac1b expression in stromal cells in normal ovary tissue, colors as for middle panel. (**B**) The majority of serous papillary ovarian adenocarcinoma cells were moderately to strongly positive for Rac1b, while stromal cells were weakly positive. (**C**) Quantitative comparisons of normal ovary tissue and serous papillary ovarian adenocarcinoma tissue evidences an elevated expression of Rac1b in the stromal cells adjacent to the malignant tumor cells relative to normal tissue.

**Figure 6:**
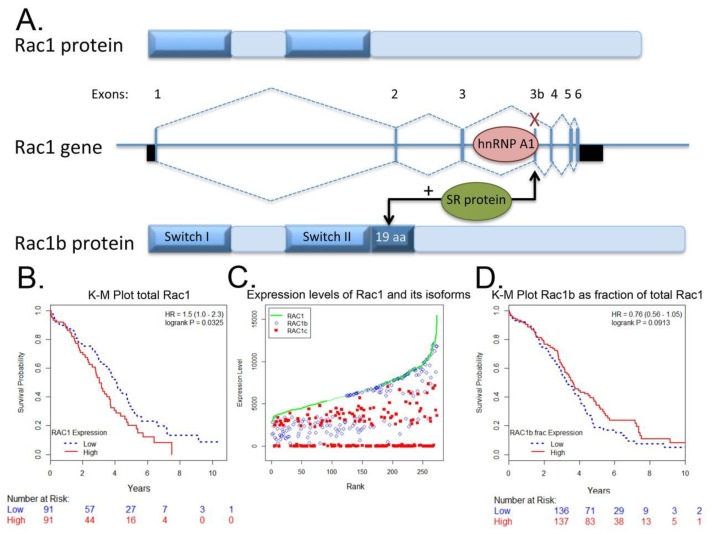
High total RAC1 expression predicts reduced ovarian cancer patient survival. (**A**) Rac1 undergoes regulated splicing in response to growth factor signaling, which is subject to positive and negative regulation by hnRNP A1 and SR protein. The resulting splice variant is called Rac1b and contains a 19 amino acid insert adjacent to the Switch II region. Rac1b is a fast cycling, constitutively active and frequently overexpressed in cancer, including ovarian cancer. (**B**) Kaplan-Meier plot of high vs. low total RAC1 mRNA expression. TCGA datasets for total and RAC1 isoform mRNA expression in ovarian cancer patients from ISOexpresso; uc003spx.3 (canonical RAC1) and uc003spw.3 (RAC1b containing exon 3b/4). Analyses were restricted to 298 patients with Stage III and Stage IV disease. Patients were divided into 3 groups based on total Rac1 expression. Upper tertile values represent high total Rac1 expression and lower tertile values represent low expression, middle values were excluded. Patients with high RAC1 expression have worse survival outcomes than those with low RAC1 expression (HR = 1.5, *p* = 0.0325); analogous results obtained using data direct from TCGA and CASViewer. No evidence for an association between isoform RAC1b and survival outcomes (HR = 0.96, *p* = 0.82, not shown). Higher expression of the canonical RAC1 isoform trended to lower survival probability, though was not statistically significant (HR = 1.37, *p* = 0.121). (**C**) Plot of total RAC1 (green line), canonical RAC1c (blue diamond), and RAC1b (red square) expression in each patient ranked according to expression levels. (**D**) Kaplan-Meier plot of RAC1b as a fraction of total RAC1, with two groups defined based on median expression. High RAC1b expression (HR = 0.76, *p* = 0.0913).

The constitutively active Rac1b splice variant mRNA level [113] and protein levels are moderate to high in the majority of serous papillary ovarian adenocarcinoma cells (Figure 5). Interestingly, Rac1b is also differentially expressed in underlying stromal cells in malignant serous papillary ovarian adenocarcinoma tissue as compared to normal ovary. The prognostic or diagnostic significance of overexpression of canonical Rac1 in ovarian cancer and/or the potential role(s) of the hyperactivated, fast cycling Rac1b isoform remain open questions. We analyzed RAC1 mRNA expression data for 298 Stage III primary serous ovarian cancer patient samples in TCGA using isoform analysis tools [154,155]. The results demonstrate that high total RAC1 mRNA expression is associated with worse outcomes (Figure 6A,B) and concur with a report that analyzed Rac1 as a risk factor in a cohort of 150 Chinese ovarian cancer patients [47]. High expression of the canonical RAC1 isoform also trended to worse outcomes but was not statistically significant (Figure 6C). The impact of RAC1b isoform expression on ovarian patient survival has not been reported and was of particular interest. Rac1b protein drives tumor cell proliferation and EMT and is upregulated by MMP3, a known survival risk factor in breast, lung, and pancreatic cancers [124,156,157,158]. High mRNA expression of the fast cycling and constitutively active RAC1b isoform does not predict ovarian cancer patient survival and trended toward higher survival probability (Figure 6D [113,120,121,122,129,130,155]); the finding was consistent irrespective of various groupings, treatment as a continuous variable or when expressed as a fraction of total RAC1 mRNA expression. The only other study assessing the significance of RAC1b isoform expression measured the prognostic value of RAC1b in progression free and overall survival [159]. Findings were based on quantitative RT-PCR analyses of 157 metastatic colorectal cancer patient samples following relapse after first line chemotherapy. In contrast to our findings in primary ovarian tumors, fractional RAC1b overexpression was significantly associated with poor progression free (HR 0.54 *p* = 0.49) and overall survival (HR 0.53, *p* = 0.039) in metastatic colorectal cancer patients. Similar to the ovarian cancer patients, RAC1b expression was not mutually exclusive and 152/157 (97%) of the metastatic colorectal patients had higher canonical RAC1 than RAC1b expression. To date there are no studies that have distinguished the functions of Rac1 and Rac1b overexpression or activity in the absence of endogenous protein, in part due to the essentiality of Rac1 function [128,160]. Together, these data indicate that overexpression and aberrant Rac1 and/or Rac1b activity are closely tied to malignant ovarian cancer and further dissection of their respective roles in tumor microenvironment responsiveness, metastasis and relapse is warranted.

## 5. Potential Benefits of Targeting Aberrant Rac1 Activity in Ovarian Cancer

The broad impact of Rac1 on tumor cell behavior has led to consideration of Rac1 as a potential therapeutic target [25,28,95,161,162,163]. In ovarian cancer cell lines, knock down of Rac1 expression decreased fibronectin production [13], reversed EMT as measured by increased E-cadherin and decreased vimentin expression [46,47], inhibited tumor cell migration and invasion [47] and reduced tumor growth in a xenograft model [47]. An inhibitor of Rac1 (NSC23766) decreased ovarian tumor cell migration, invasion and matrix-metalloproteinase production [48,49,95].

Although a number of small molecule inhibitors have been developed to inhibit Rac1 activity (e.g., NSC23766, EHT 1864, EHop-016 and its derivative MBQ-167), these agents have not been translated to human use [66,164,165,166]. A high-throughput screen of the Prestwick library of off patent, FDA-approved drugs identified activators and inhibitors of Rho GTPases [95]. The resultant findings coupled with cheminformatics approaches identified the R-enantiomers of a limited number of non-steroidal anti-inflammatory drugs (NSAIDs), R-naproxen and R-ketorolac, as inhibitors of Rac1 [95]. The S-enantiomers are pharmacologic NSAIDs based on cyclooxygenase (COX) inhibition. GTPase inhibition by the R-enantiomers represents a previously unidentified pharmacologic activity. R-naproxen and R-ketorolac inhibit serum and EGF-stimulated Rac1 and Cdc42 activation and downstream signaling through a proposed allosteric mechanism [48,95]. R-ketorolac was tested using ovarian tumor cell lines and primary ovarian tumor cells isolated from patient ascites fluids [48]. R-ketorolac was an effective Rac1 inhibitor and decreased downstream signaling as demonstrated by reduction of PAK1 and PAK2 phosphorylation. R-ketorolac inhibited Rac1-dependent cellular functions in ovarian cancer cell lines and primary cells including inhibition of growth factor-stimulated formation of filopodia, cell adhesion to fibronectin and type I collagen, development of invadopodia and tumor cell migration [48]. The inhibitory effects of R-ketorolac in cells are comparable to those of established Rac1 and Cdc42 selective inhibitors [48,167].

In Phase 0 human studies, ovarian cancer patients received racemic ketorolac for its FDA-approved indication in postoperative analgesia [113] then blood and peritoneal fluids were collected at intervals for 24h. After administration of the racemic drug, R-ketorolac was detected in patient peritoneal fluids. The concentration of R-ketorolac was sufficient to inhibit Rac1 activity in cells retrieved from the peritoneal compartment of these post-surgical ovarian cancer patients. Potential benefit of R-ketorolac is suggested by the results of a medical record review to compare the ovarian cancer–specific survival of ovarian cancer patients who did or did not receive ketorolac [113]. The medical record review revealed increased survival of patients receiving ketorolac and this observation is consistent with other reports of improved clinical outcomes associated with ketorolac usage in breast cancer patients [168,169,170]. The overall findings suggest that ovarian cancer patients may benefit from inhibition of Rac1 in the clinical setting.

## 6. Other Ovarian Tumor Microenvironments: Extraperitoneal Dissemination and Bone Niche as a Sanctuary Site and Potential Reservoir for Relapse

While ovarian cancer metastasis is largely confined to the peritoneal cavity and localized to the omentum, there is strong evidence for extra-peritoneal dissemination [171,172]. As illustrated in preceding sections, Rac1 plays a critical role in the key processes that impact tumor dissemination and as such, Rac1 may contribute to ovarian tumor cell escape from the peritonium. Particularly in advanced disease, ovarian carcinoma can spread to distant organs by both hematogenous dissemination and lymphatic invasion [173]. In a well-designed parabiosis study, ovarian tumor cells were found to spread in anastomosed mice within two weeks of ovary injection [18], clearly illustrating hematogenous spread of the disease. Additionally, circulating tumor cells (CTCs) are frequently detected in patients [53,174]. In fact, CTCs were detected in 90% (98/109) of newly diagnosed ovarian cancer patients, where the number of CTCs correlated with disease stage and was altered with treatment [175]. Lymph node involvement of the disease is also common and has been proposed as a potential prognostic factor with site-specific prognostic differences identified between the ovary and lymph node [176]. However, this study was unable to rule out the “safe haven” hypothesis for metastatic ovarian tumor cells in retroperitoneal lymph nodes and suggested that lymph node dissection after complete cytoreduction is warranted pending further prospective data collection [176]. Interestingly, recent work comparing the survival of patients with distant lymph node metastases to patients with pleural metastases or other distant ovarian cancer metastases found increased survival in women having lymph nodes as their only distant metastatic site [177]. A follow up study investigating the relationship between site-specific patterns of distant metastases and overall survival also found that patients with lymph node metastasis had the longest survival when compared to women with other metastatic disease [173]. Collectively, these data suggest that disease dissemination through the lymphatics may have a less aggressive phenotype than disease that spreads hematogenously. Future studies will be necessary to quantitatively compare the aggressive nature of ovarian cancer cells with respect to their route of disease dissemination.

Once outside the peritoneum, other common sites of distant metastatic ovarian cancer include the liver, lung, and bone [6]. While frank bone metastases are rare in ovarian cancer [173,178], prognosis of cases with bone metastasis is poor. A recent publication [179] followed up on previous observations of bone marrow disseminated tumor cells (DTCs) in ovarian cancer patients [51,52,180,181] and affirmed that bone DTCs correlated with reduced progression free and overall survival [182,183]. Bone marrow was isolated from 79 ovarian cancer patients pre- and post-platinum-based chemotherapy. Bone DTCs were detected in 42% and 41% of patients before and after chemotherapy, respectively, illustrating the chemoresistance of cells in the bone niche [179]. Alterations in the bone microenvironment caused by irradiation and cisplatin therapy can further promote and increase metastatic spread that may be ameliorated by non-steroidal anti-inflammatory agents [184]. Additionally, tumor secreted factors such as CCL2 can activate cells in the bone marrow promoting a premetastatic niche and paving the way for successful tumor dissemination at a secondary site [98]. The predominant signaling axis that promotes bone marrow homing is the CXCR4/SDF-1α signaling cascade [185]. The expression and secretion of SDF-1α is abundant in the bone marrow microenvironment (expressed by osteoblasts and endothelial cells) and promotes the homing and maintenance of CXCR4+ cells within the bone marrow. In addition to driving hematopoietic cells as well as breast and prostate cancer cells to the bone [186,187,188,189], CXCR4/SDF-1α signaling has also been shown to promote ovarian cancer metastasis and is a predictor of poor prognosis in ovarian cancer [190,191]. Overexpression of CXCR4 is associated with cisplatin resistant ovarian cancer [192] as well as the peritoneal [193], hematogenous [194] and lymph node [195] dissemination of the disease. Moreover, CXCR4 can modulate cancer cell migration through interactions with the downstream effector Rac1 [196]. In fact, blocking or silencing of CXCR4 was found to significantly reduce RhoA and Rac-1/Cdc42 expression levels and decrease ovarian cancer cell migration [197]. Additionally, CXCR4 blockade reduced ovarian tumor growth in animal models [198,199]. Therefore, CXCR4 appears to be a shared signaling mechanism that facilitates homing and engraftment within the peritoneal cavity and the bone marrow microenvironment. How Rac1 specifically influences ovarian tumor cells within these two separate environments remains to be explored.

Ovarian cancer metastasis has long been studied in the context of the peritoneal compartment where the bulk of the tumor grows. However, as we improve our systemic and palliative therapy for ovarian cancer patients, an increasing occurrence of unusual distant metastases is being reported. Despite compelling human findings, the overall significance of the bone niche with respect to ovarian cancer prognosis remains ill-defined and suggests that a shift in research focus to understudied metastatic sites such as the bone will be critical to improving patient outcomes. Moreover, the bone marrow dissemination of ovarian cancer cells has been largely overlooked as a potential mechanism for relapse, where the persistence of tumor cells in the protected bone niche could contribute to disease recurrence. Therefore, future studies should be directed at identifying factors that enable tumor cells to be harbored in specialized niche sites that include the bone. By targeting bone marrow-resident tumor cells, we may uncover mechanistic strategies to eradicate distant tumor cell reservoirs that contribute to ovarian cancer relapse and poor overall patient survival.

## 7. Conclusions

Ovarian cancer remains a leading cause of death in women resulting from gynecologic malignancy principally due to recurrent, drug resistant disease, and limited options for targeted therapies. Greater understanding of signaling proteins that mediate tumor microenvironmental drivers of disease and resistance may provide new avenues for therapeutic development. Rac1 is at the nexus of numerous signaling pathways stimulated by the ovarian cancer TME and has broad roles in cancer beyond the well-recognized regulation of actin remodeling, tumor cell adhesion and migration. Rac1-dependent functions in EMT, stem cell phenotypes, angiogenesis and chemoresistance all have high relevance to ovarian cancer. Although more research is needed regarding specific contributions of aberrant Rac1 activity in ovarian cancer and disease dissemination with respect to specialized microenvironments, current knowledge suggests benefits of targeting Rac1, alone or in combination, for disease treatment.
